# Oligomeric Structure of the MALT1 Tandem Ig-Like Domains

**DOI:** 10.1371/journal.pone.0023220

**Published:** 2011-09-23

**Authors:** Liyan Qiu, Sirano Dhe-Paganon

**Affiliations:** 1 Structural Genomics Consortium, University of Toronto, Toronto, Ontario, Canada; 2 Department of Physiology, University of Toronto, Toronto, Ontario, Canada; University of Cambridge, United Kingdom

## Abstract

**Background:**

Mucosa-associated lymphoid tissue 1 (MALT1) plays an important role in the adaptive immune program. During TCR- or BCR-induced NF-κB activation, MALT1 serves to mediate the activation of the IKK (IκB kinase) complex, which subsequently regulates the activation of NF-κB. Aggregation of MALT1 is important for E3 ligase activation and NF-κB signaling.

**Principal Findings:**

Unlike the isolated CARD or paracaspase domains, which behave as monomers, the tandem Ig-like domains of MALT1 exists as a mixture of dimer and tetramer in solution. High-resolution structures reveals a protein-protein interface that is stabilized by a buried surface area of 1256 Å^2^ and contains numerous hydrogen and salt bonds. In conjunction with a second interface, these interactions may represent the basis of MALT1 oligomerization.

**Conclusions:**

The crystal structure of the tandem Ig-like domains reveals the oligomerization potential of MALT1 and a potential intermediate in the activation of the adaptive inflammatory pathway.

**Enhanced version:**

**This article can also be viewed as an enhanced version in which the text of the article is integrated with interactive 3D representations and animated transitions. Please note that a web plugin is required to access this enhanced functionality. Instructions for the installation and use of the web plugin are available in Text S1.**

## Introduction

**Figure 1:**
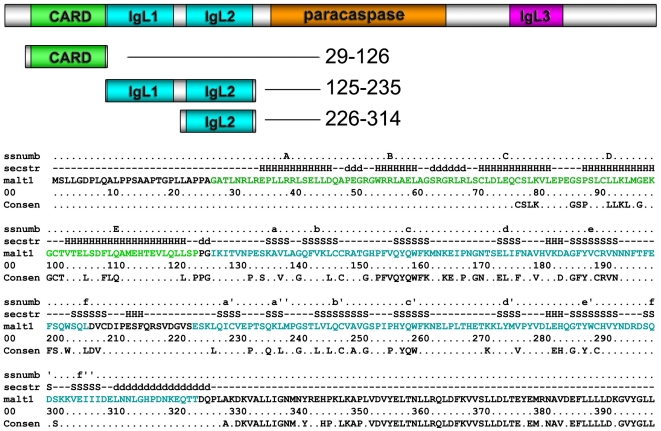
MALT1 domain architecture and sequence details. Domain schematic is shown above. Diagram of MALT1 CARD, tandem IgL1–IgL2 and IgL2 domains are shown in the middle. Secondary structure labels (ssnumb), secondary structure elements (secstr; H = helices, S = strands, D = disordered), primary sequence (malt1), sequence numbering (00), and phylogenetic sequence conservation (Consen) are shown at the bottom. Domains are highlighted in different colors. Helices are labeled in upper case letters and strands are labeled in lower case letters.

Mucosa-associated lymphoid tissue (MALT) lymphoma is a low-grade tumor composed mainly of B-cells characterized by chronic inflammation [1], [2]. Many of these tumors reside within the stomach epithelium [3]. A subset of MALT lymphomas are caused by genetic translocation events that result in fusion proteins of the N-terminal region of cIAP2 and the C-terminal region of MALT1. Wild-type cIAP2 contains tandem baculovirus IAP repeat (BIR) domains followed by a ubiquitin-associated (UBA) domain, Caspase recruitment (CARD) domain and Really Interesting New Gene (RING) domain. Wild-type MALT1 contains a CARD-like death, three Ig-like, a paracaspase domain (**Figure 1**). Translocation occurs immediately after the cIAP2 UBA domain and either just before the first Ig-like domain, the second Ig-like domain, or the paracaspase domain. Resultant adducts chronically activate the inflammatory NF-κB signaling pathway and predispose or cause disease [4]. How the resultant fusion protein activates NF-κB to cause low grade inflammation in disease remains unclear.

The biological role and function of MALT1 is related to the adaptive immune response, playing an important role in signal transduction, specifically in antigen B-cell receptor activation [5]. MALT1 contributes upstream in the inflammatory pathway, activating E3 ligases (TRAF2/6) that are normally used by the innate immune response to activate the IKK and TAK kinase complexes, which directly regulate transcription factors NF-κB and cJUN, respectively. How MALT1 activates the E3 ligases (TRAF2 and 6) remains unclear.

Activation of many E3 ligases is associated to their oligomerization or aggregation state, but the precise mechanism of activation is unclear [6], [7]. Clustering of TRAF2/6 is thought to depend on aggregattion of the CMB complex, which is composed of CARMA1, MALT1, and Bcl10. Clustering of this complex is dependant on phosphorylation, particularly by PKC isoforms beta and theta, induced by the canonical phospholipase signaling pathways activated by B-cell receptors [8], [9], [10]. Phosphorylation of CARMA1 nucleates the multiprotein complex and recruits the enzyme TRAF2/6. Substrates of TRAF2/6 complexes are diverse, and likely include itself and IKKγ. Interestingly, autoubiquitylation may create binding sites for IKKγ. Ubiquitylation of the latter in turn is important for activation of IKKβ and for NF-κB activation. The mechanism through which IKKβ is activated is unclear.

The CMB complex is held together by many points of interaction, including an interface between the MALT1 death and tandem Ig-like domains with Bcl10 [11], [12], contact between CARMA1 and the C-terminus of MALT1, and an association between TRAF2/6 and a C-terminal segment of MALT1. Yet, it is elusive how the CMB complex clusters or oligomerizes. Potentially, signal-induced phosphorylation of CARMA1 initiates clustering through its oligomer-prone CARD domain [13].

The death domain (DD) superfamily comprises of the following subfamilies: the death domain (DD), the death effector domain (DED), the caspase recruitment domain (CARD), and the pyrin domain (PYD). CARD domains participate in the assembly of oligomeric signaling complexes by mediating homotypic interaction with other DD superfamily proteins [14]. They are involved in apoptosis through their regulation of caspases that contain CARDs, including human caspases 1, 2, 8, 9 and 10, which could promote apoptosis through proteolytic degradation of other cellular components [15], [16], [17]. CARDs are also involved in inflammation through their regulation of NF-κB activation in TNF signaling. The mechanisms by which CARDs activate caspases and NF-κB involve the assembly of oligomeric platforms, which can facilitate dimerization or serve as scaffolds on which proteases and kinases are assembled and activated [14], [15], [16]. Although speculative, the complex may be stabilized by heterotypic interactions between the CARD and CARD-like domains of CARMA1, MALT1, and Bcl10.

Clustering mechanisms of signaling molecules are the basis of adaptive and innate immune signal transduction. And these mechanisms are likely the molecular basis of how cIAP2-MALT1 fusion proteins activate the inflammatory pathway without upstream activators. In order to better define the oligomerization potential of MALT1, we solved the structure of the MALT1 N-terminal CARD-like death domain and the tandem Ig-like domain. Here we show using structural biology that unlike the CARD-like death domain, the tandem Ig-like domains naturally form oligomers with a tendency towards dimers and tetramers. Structures reveal the molecular basis of dimerization and tetramerization by the Ig domain and suggest that MALT1 oligomerization may be mediated at least in part by its tandem Ig-like domains. This study may help understand how MALT1 acts as an oligomeric scaffold protein to bind co-factors like Bcl10, CARMA1, and TRAF6, and activate the NF-κB pathway.

## Results and Discussion

### CARD domain

**Figure 2:**
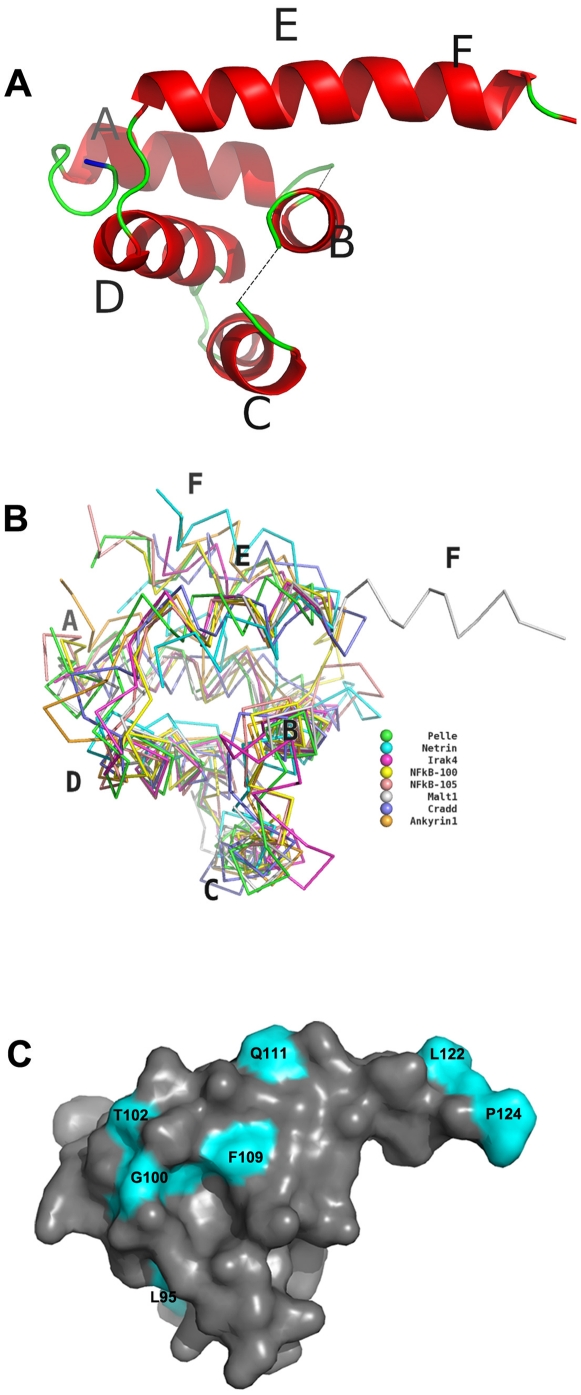
Structure of the MALT1 CARD domain. (**A**) Ribbon representation of the MALT1 CARD domain. Alpha-helices are labeled; α-helices E and F are contiguous in MALT1. (**B**) Alignment and backbone representation of CARD domains, including MALT1, serine/threonine-protein kinase Pelle, Interleukin-1 Rak-4, Netrin Receptor, Ankyrin-1, Cradd, and NF-kB P100/105, which were identified by a Dali search having Z scores greater than 10. (**C**) Surface representation of the MALT1 CARD domain is shown with strictly conserved residues labeled in cyan.

The human MALT1 N-terminal CARD domain (residues 29–126) was 1) cloned into a pET28 vector system, 2) overexpressed in transformed BL21 bacteria, 3) purified using IMAC and size-exclusion chromatography, and 4) crystallized in potassium tartrate. Its structure was determined using a selenomethionine derivative (Table 1). It is composed of a five-helix bundle; the four and six-residue A–B and B–C loops, respectively, were disordered (**Figure 2A**). A Dali search using the MALT1 CARD domain revealed six structures in the Protein Data Bank (PDB) with Z scores greater than 7.0, including serine/threonine-protein kinase Pelle, Interleukin-1 Rak-4, Netrin receptor, Ankyrin-1, Cradd and NF-kB P100 [17]. Their CARD crystal structures were superimposed with that of MALT1 (**Figure 2B**); the rmsd (root mean squared deviation) between the MALT1 and the individual proteins was 1.4, 1.9, 2.2, 2.2, 2.3 and 4.9, Å, respectively, despite sequence similarities, over the aligned regions, less than 25% (data not shown). Therefore, except for the last helix, the MALT1 topology corresponds to the caspase recruitment domain (CARD). The CARD domain is a protein-protein interaction module, typically associating with itself or other CARD-containing proteins, forming either dimers or trimers [18], [19], . In contrast to canonical CARD domains, the sixth (F) helix in MALT1 is continuous with the fifth (E), forming a long helix that extends beyond the globular core of the domain (**Figure 2A**).

**Table 1:** 

Diffraction Data and Refinement Statistics			
Domain	CARD	IgL1–IgL2	IgL2
Residue range	29–126	129–326	226–314
PDB code	2G7R	3K0W	3BFO
Beamline	APS 19-BM	CLS 08ID-1	CHESS F1

Diffraction Data			
Space group	C2	P6_2_22	P1
Resolution (Å)	19.8-2.70	40.50-2.8	25.0-1.15
Highest resolution shell	2.77-2.70	3.08-2.80	1.18-1.15
Cell dimensions: a, c (Å)	86.937, 34.598	90.355, 182.173	39.944, 49.595
Unique Reflection	6562	11498	103829
Completeness (%)#	94.6 (91.8)	99.9 (91.8)	94.6% (91.8)
Redundancy#	1.8 (1.9)	18.8 (17.9)	5.1 (3.70)
Rsym (%)	13	8	6
I/sigI#	18 (4.0)	43.44 (2.80)	22.0 (2.48)

Model refinement			
Resolution (Å)	19.80-2.70	40.47-2.8	25-1.15
All Atoms (solvent)	1333 (3)	1472 (15)	3441 (401)
Rwork/Rfree %*	23.6/28.4	21.83/24.49	17.77/21.2
Rfree calculated from the data (%)	4.4	5.2	5.0
Rmsd bond lengths/angles	0.012/1.565	0.010/1.32	0.017/1.83
Mean B-factor (Å^2^)	24.6	87.14	19.98
Ramachandran MolProbity favored/outliers (%)	98.6/0	92.86/0	98.6/0

# : value for highest resolution shell shown in parenthesis.

The unusual position of the sixth helix is also found in the crystal structures of another CARD containing protein, NOD1, where it is involved in a domain-swapping mechanism. The sixth helix sits in its canonical position, between helices A and E, but in another molecule. This was suggested to mediate homodimerization of NOD1. Unfortunately, the presence of a disulfide bond between the molecules clouded the conclusions [18], [19]. The sixth helix of MALT1 CARD domain, in the crystalline state, also sits in its canonical position in another molecule, but not in a swapped dimer interaction. Instead, the helix is involved in a head-to-tail interaction with a neighboring molecule. Consistent with a lack of noticeable homodimeric interactions in the MALT1 crystalline state, a PISA analysis of the MALT1 CARD domain revealed no significant indications of assembly formation in solution [20]. Furthermore, our size-exclusion analysis showed a monomeric behavior (**see subsequent sections**). Taken together, this suggested that instead of homodimerization, the CARD domain might be competent to heterodimerize.

CARD-containing MALT1 binding partners include Bcl10 and Caspase 8 [21]. The interaction with Bcl10 was loosely mapped to the N-terminal region of MALT1 [11]. Although the precise mode of interaction was unclear, the CARD domains contributed but were insufficient for interaction. A direct interaction between MALT1 CARD and Bcl10 CARD could not be detected by co-immunoprecipitation, but FRET assay and mutagenesis analysis showed the CARD domain of Bcl10 and MALT1 also contribute to the Bcl10-MALT1 interaction.

The surfaces of CARDs are usually polarized with basic surfaces on one side and acidic on the other; an observation that has led some to suggest that this is the basis of putative protein-protein interactions [15]. To evaluate a potential CARD-CARD interaction between MALT1 and Bcl10, we constructed using the 3D-JIGSAW server a 3-dimensional model of the human Bcl10 CARD domain (residues 18–102) using RAIDD as a template (**Figure S1**). As with MALT1, the Bcl-10 CARD model adopted a polarized charged surface, with helices B, E and F being acidic, and helices A, C, and D (the opposite face) being basic. These complementary surface charges may mediate heterodimerization, but further verification undoubtedly would be required. Unfortunately, nothing could be gleaned from phylogenetic analyses, because although the amino acid sequence of the last three helices of the MALT1 CARD domain was conserved, almost all of the conserved residues occurred in the core of the domain; in other words, no large surface-exposed patch of the domain was conserved (**Figure 2C**).

### First Ig-like domain (IgL1)

**Figure 3:**
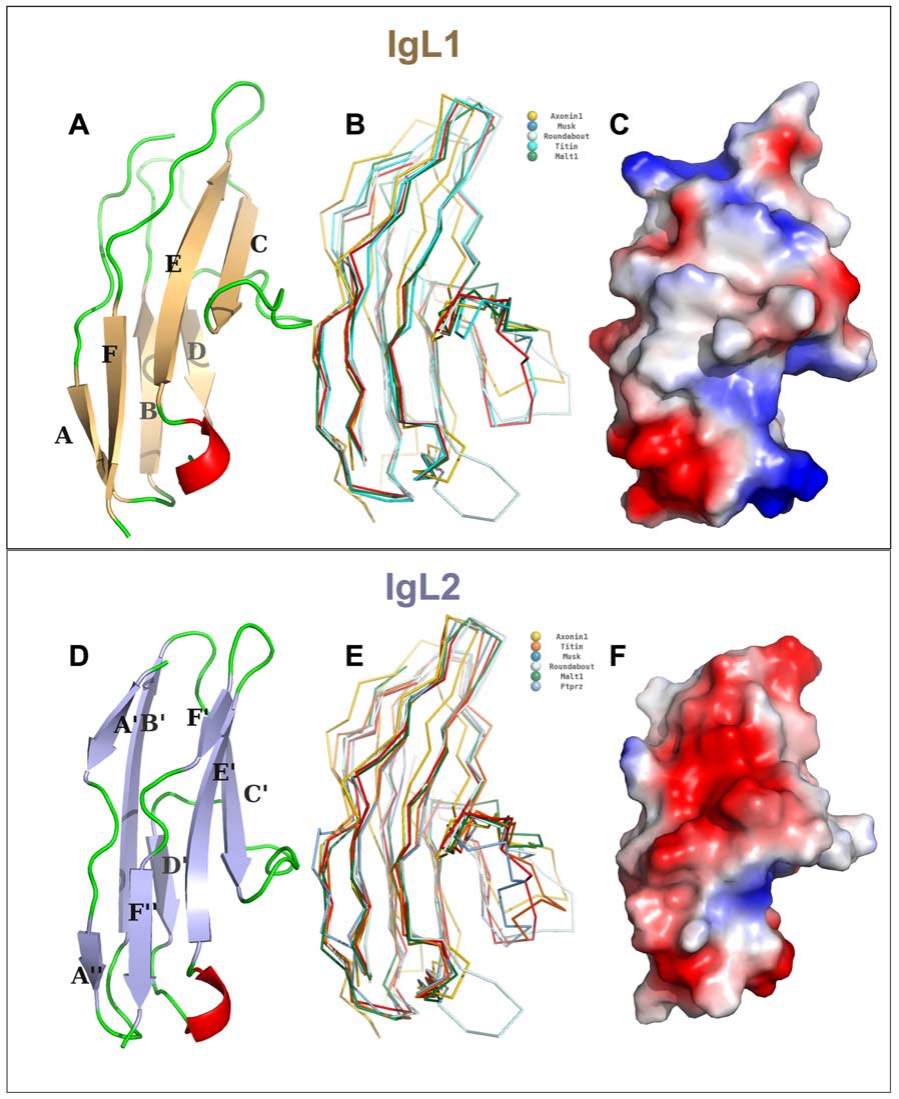
Structure of the IgL1 and IgL2 domains. Ribbon representations are shown for IgL1 (**A**) and IgL2 (**D**) domains, with β-strands labeled. Alignment of superimposed homologous structures of MALT1 IgL1 (**B**) and IgL2 (**E**) domains, identified by Dali search having a Z score greater than 10.0, are shown with different colors as backbone representations. Shown are electrostatic surface representations with a gradient from −10 (red) to 10 (blue) kT/e of MALT1 IgL1 (**C**) and IgL2 (**F**) domains.

Although sufficiently soluble on its own, we were unable despite extensive screening, to crystallize the first Ig-like domain. But together with the second Ig-like domain, the tandem construct was crystallized and diffracted well (Table 1). The structure of the first MALT1 Ig-like domain revealed the classical sandwich-like Ig fold (about 80–100 amino acids long) [22], formed by two antiparallel β-sheets [23], [24] (**Figure 3A**); β-strands A, F, E, and C formed one sheet, and B and D formed the other (**Figure 3A**). Strand A contained a β-bulge; β-turns and β-hairpins are found at each extremity of the β-sandwich structure of the tandem Ig domains. Only β-strands A and B were connected by a short β-loop (2 to 5 residue loop), while the others were connected by longer loops (8 to 11 residues loop). A 3–10 helix connected strands D and E. The hydrophobic core of the N-terminal domain was dominated by residues Ala137, Val138 from β-strand A, Val144, Leu146 from β-strand B, Trp160 from β-strand C, Leu175, Ile176 from β-strand D, Tyr188 from β-strand E and residues Leu206, Val208 from β-strand F. Although many Ig-like domains have within their cores buried disulfide bonds, the MALT1 Ig-like domains, despite having a pair of very closely associated cysteine residues, with their γ-sulfur atoms 4.0 Å apart, did not support disulfide bridges. Although almost without exception intracellular proteins have evolved to be stable in the absence of these bridges, the close proximity of these residues in the core of these domains is intriguing.

A Dali search using the MALT1 IgL1 domain revealed four structures in the Protein Data Bank (PDB) with Z scores greater than 10, including Musk (Z-score = 12, rmsd of 1.8 Å for 82 aligned Cα, 27% sequence identity), Axonin1 (Z-score = 10.7, rmsd of 2.7 Å for 87 aligned Cα, 23% sequence identity), Roundabout1 (Z-score = 11.5, rmsd of 2.8 Å for 89 aligned Cα, 21% sequence identity) and Titin (Z-score = 11.2, rmsd of 2.3 Å for 83 aligned Cα, 25% sequence identity) (data not shown). Variations in loop structures occured almost exclusively at the C–D and D–E loops (**Figure 3B**). Interestingly, these loops formed the walls of the largest cleft of the domain with a phylogenetically conserved hydrophobic pocket and hydrophilic perimeter that may represent a binding site for a heretofore-unknown ligand (**Figure 3C**).

### Second Ig-like domain (IgL2)

The second MALT1 Ig-like domain (IgL2) was crystallized by itself and as a tandem domain with IgL1 (Table 1). No conformational differences were observed in these two contexts (data not shown). IgL2 was similar to IgL1, with an rmsd of 1.6 Å over 86 Cα atoms and a 27% sequence identity, the bulk of which was mapped to the domain core (data not shown). Unsurprisingly, a Dali search using the IgL2 structure identified the same set of proteins as before, including Musk, Ptpr2, Titin, Axonin1, and Roundabout1 with Z scores greater than 12.0, an rmsd of 1.2–1.8 Å over about 82 Cα atoms, and an average of 25% sequence identity (data not shown). Similar to IgL1, variations in loop structures occurred almost exclusively at the C–D and D–E loops (**Figure 3E**), forming the largest cleft of the domain (**Figure 3F**).

**Figure 4:**
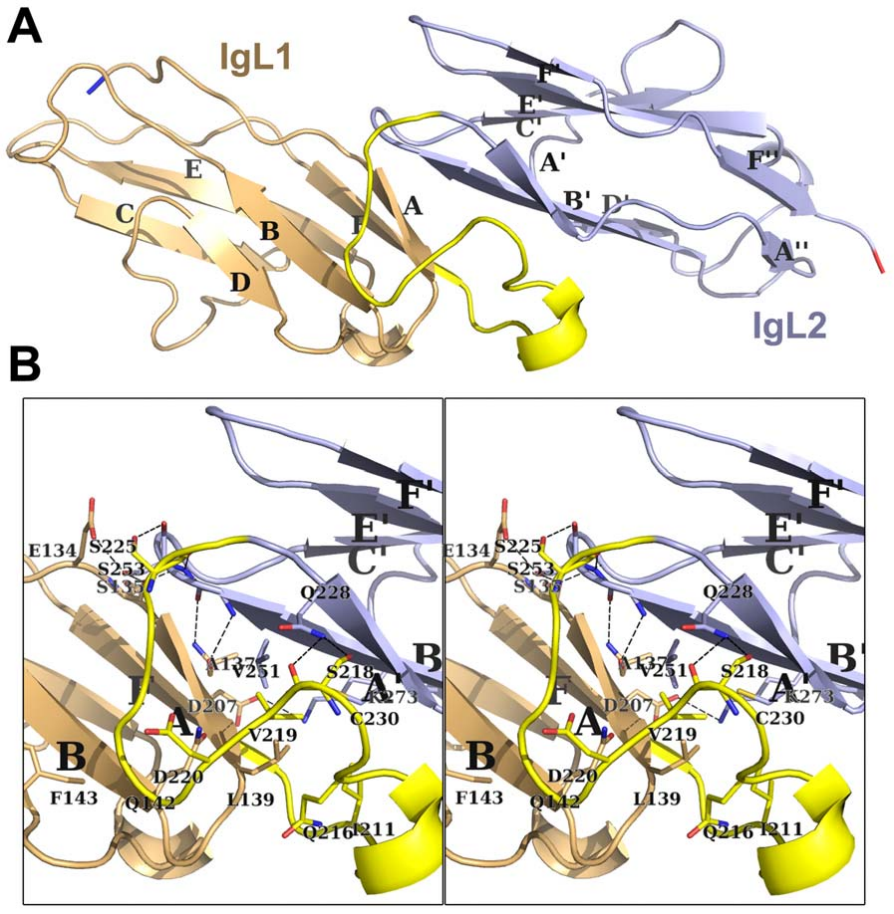
The connectedness of the tandem Ig-like domains. (**A**) Shown is a ribbon representation of the tandem Ig domains, with the first Ig domain (IgL1) in lightorange and the second (IgL2) in lightblue; the interdomain linker is shown in yellow, and α-helices are labeled. (**B**) Stereoscopic view of the interactions and buried aliphatic core between IgL1 and IgL2 is shown, with labeled secondary structures in ribbon format and side chains in line format.

The two Ig domains were arranged in a head-to-tail fashion, nearly parallel to each other, with a stagger and overlap that brought β-strand A near β-strand B' (**Figure 4A**). The domains were connected to each other via an 18-residue loop containing a 3–10 helix that buried a hydrophobic core derived from β-strands A, A', and B' (**Figure 4B**). In addition, seven hydrogen bonds and a salt bridge formed at the interface between the two domains (**Figure 4B**). The large number of inter-domain interactions and the value of the B-factors at this interface being similar to the core residues of the individual domains collectively suggested that the two domains remain fixed relative to each other.

Many Ig domains appear to function as insulators, structural elements that keep adjoining domains apart from each other. If this is the case with MALT1, the tandem IgL domains insulate their adjoining domains by a space of 67 Å, the distance between the N-terminal residue of IgL1 (Ser127) and the C-terminal residue of IgL2 (Glu310). The reason for keeping the CARD and catalytic domains apart from each other would be unclear.

### Oligomeric state of the tandem Ig domains

**Figure 5:**
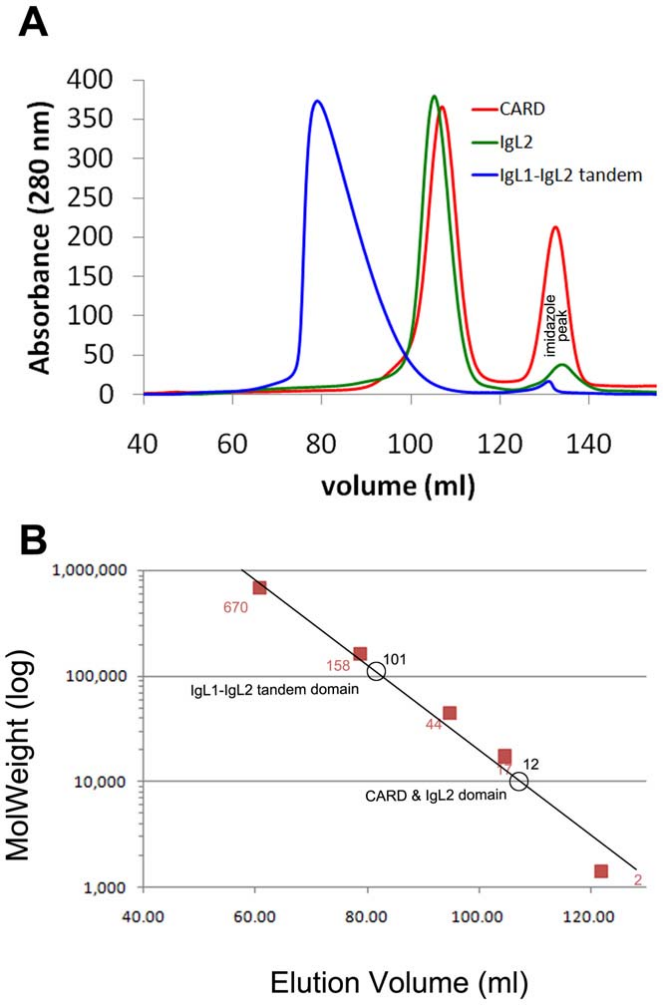
Oligomeric state analysis of the tandem Ig-like domains. (**A**) Shown are the Superdex-200 16/60 (GE healthcare) size exclusion chromatography profiles of the CARD domain, IgL2, and tandem Ig-like domains at 10 mg per ml concentration in 20 mM Tris buffer, pH 8.0, 500 mM NaCl, 1 mM tris-(2-carboxyethyl)-phosphine hydrochloride (TCEP). (**B**) Estimation of the molecular mass of CARD domain, IgL2, and IgL1–IgL2 tandem domains by gel filtration. The semi-logarithmic plot of elution volume (ml) versus molecular weight (log) of standard proteins thyroglobulin (670 kDa), globulin (158 kDa), conalbumin (75 kDa), ovalbumin (44 kDa), anhydrase (29 kDa), myoglobin (17 kDa), and ribonuclease (13.7 kDa) was shown as the standard curve. The molecular mass of the CARD domain, IgL2 and IgL1–IgL2 tandem domains (open circles) were estimated from the standard curve based on their elution volume and the molecular weights of the standard proteins (filled squares).

To explore the possibility that the N-terminal domains of MALT1 may adopt oligomeric states that would contribute to E3 ligase activation, we examined their intermolecular interaction in solution. Pure CARD and tandem IgL domains were analyzed by size-exclusion chromatography. Unlike the separate CARD and IgL2 domains, which ran as monomers, the tandem IgL1–IgL2 domains with theoretical molecular weight 23 KDa, ran as a sharp peak with an average estimated molecular weight corresponding to a tetramer (around 101 KDa) (**Figure 5A and 5B**). To validate this observation, we conducted analytical ultracentrifugation studies for the tandem IgL1–IgL2 domains, showing that the protein ran as a mixture of dimers, trimers, and tetramers (**Figure S2**). The plots of ln (abs) vs the radius square were not linear and showed an upward trend, confirming that the sample had a significant level of intermolecular interaction. The global self-association was fit and the deduced average molecular weight was 75,940 kDa. The ratio of the average observed MW to the calculated MW was 3.5: 1.

**Figure 6:**
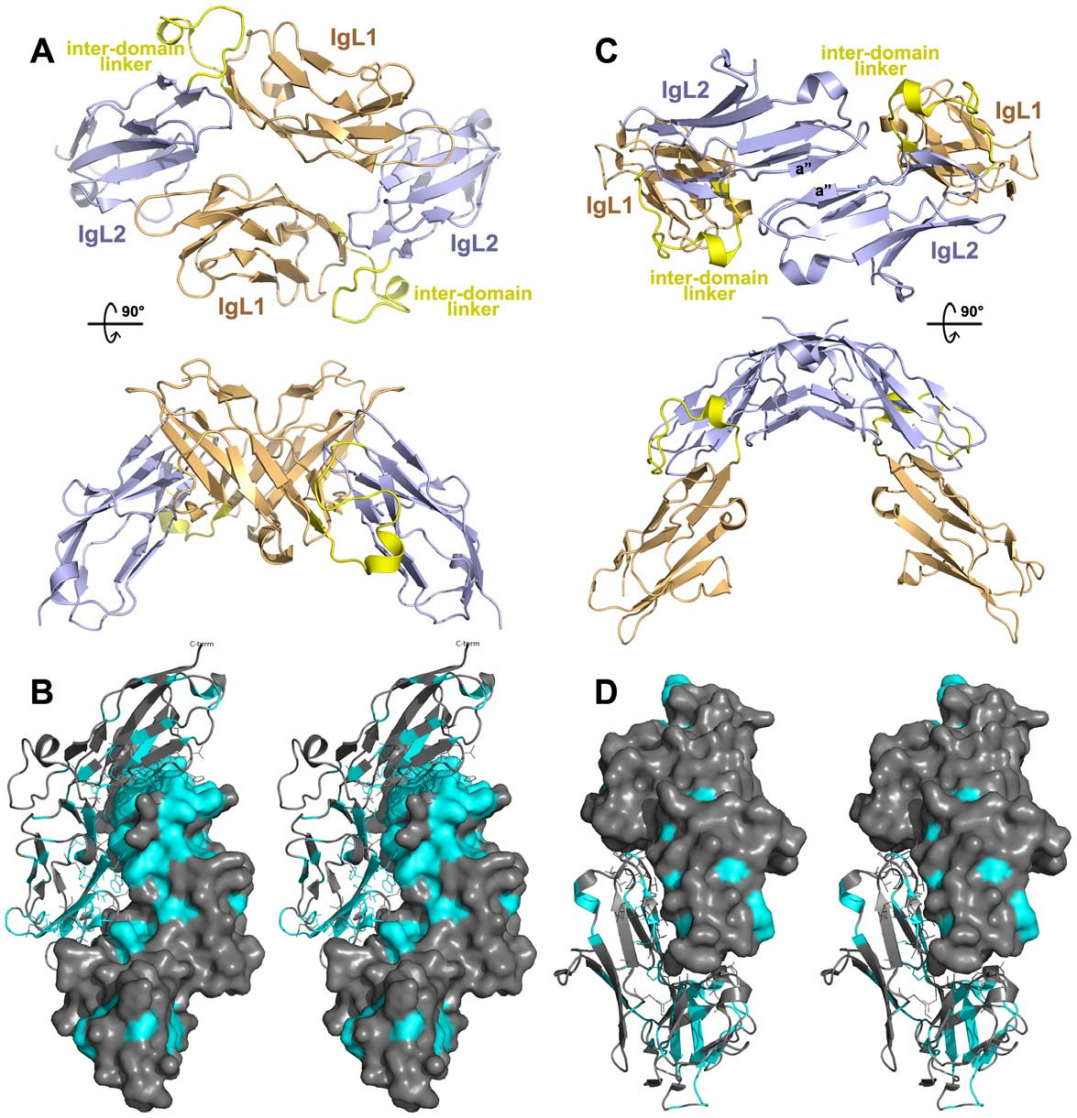
Dimeric MALT1 tandem Ig-like domains. (**A&C**) Cartoon representation of the dimeric structures of the tandem Ig-like domains. Colors are as in Figure 4 and domains are labeled. (**B&D**) Stereoscopic view of one tandem Ig-like domains shown with helices in ribbon format bound to the other Ig-like domain shown in surface format.

To examine how MALT1 might oligomerize, crystal packing and higher-order associations were examined using the PISA server (Protein Interfaces, Surfaces and Assemblies) [20]. Two quaternary interactions with CSS (Complexation Significance Score) values greater than 0.3 were found. The first buried a surface area of 1127 Å^2^ and was stabilized by 12 hydrogen and 2 salt bonds. In this assembly, two molecules were anti-parallel to each other in a cross-braced form (**Figure 6A**). The N-terminal IgL1 domain of one molecule nestled itself into a groove formed between the two domains in another molecule. Importantly, with only a few exceptions, all strictly conserved residues other than those buried in the core of the domains were buried by this quaternary assembly (**Figure 6B**). In this dimer assembly, IgL1 made most of the intermolecular interactions. The second assembly buried 670 Å^2^ surface area and was stabilized by 6 hydrogen bonds (data not shown). In this assembly, IgL2 made all interactions, the most notable of which was a joining of β-sheets through the a″ β-strands in an anti-parallel fashion (**Figure 6C**). Few surface-exposed residues of IgL2 were strictly conserved, and this assembly buried only two of these residues (**Figure 6D**). In this dimer assembly, the IgL2 made most of the intermolecular interactions. In both dimer assemblies, the center of the buried surface was predominantly aliphatic and un-charged; the surface of the first assembly was surrounded by complimentary surface charges (**Figure S3**). Together these observations support a biologically relevant role for these interactions.

**Figure 7:**
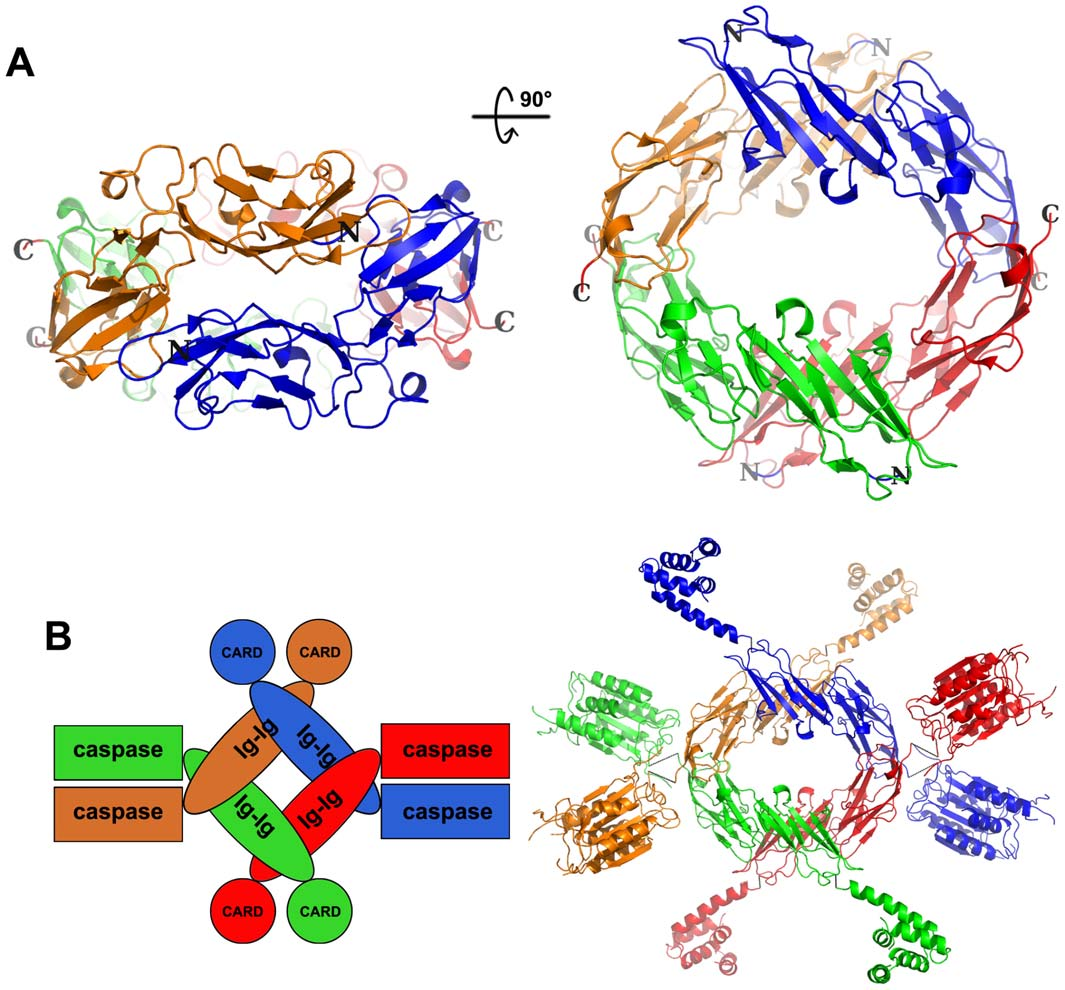
Model of oligomerization of MALT1. (**A**) Ribbon representation of the doughnut-shaped tetramer is shown from side and top views, with a 90° rotation around the horizontal axis, and with the four domains differentially colored. (**B**) Shown are schematic (left) and ribbon (right) models of a MALT1 homo-tetramer, with each molecule differentially colored and shaped (circle: CARD domain, ellipse: tandem Ig domains, and rectangle: catalytic domain).

Interestingly, if both assemblies co-exist in the same complex, an interlaced doughnut-shaped tetramer is formed (**Figure 7A**) that might represent the architectural basis of the protein's oligomerization state in solution. In this configuration, a pair of CARD domains would be near each other as would a pair of catalytic domains. A three-dimensional model of MALT1 was generated using the HEX interactive protein docking and molecular superposition program and provided a glimpse into a putative atomic model of an oligomeric MALT1 (**Figure 7B**). The following signaling mechanism is proposed: upstream activation-signals cluster CARMA1 and MALT1, which in turn cluster and activate associated E3 ligases.

## Materials and Methods

### Cloning, protein expression, and purification

The coding region of MALT1 CARD (residue 29–124), tandem Ig domains (residue 127–310), and IgL2 domain (residue 225–309) were cloned using a Mammalian Gene Collection cDNA template (AU79A6) into the pET28a-LIC vector (GenBank) using the In-Fusion CF Dry-Down PCR Cloning Kit (Clontech). Competent BL21 (DE3) cells (Invitrogen) were transformed and grown using the LEX system (Harbinger BEC) at 37°C in 2 L bottles (VWR) containing 1800 ml of TB (Sigma) supplemented with 150 mM glycerol, 100 µM Kanamycin and 600 µl antifoam 204 (Sigma). At OD (600) = 6.0, the temperature was reduced to 15°C, and one hour later the culture was induced with 100 µM IPTG and incubated overnight (16 hours) at 15°C. Cell pellets were resuspended in 30 mL per liter bacterial culture of lysis buffer (20 mM Tris pH 8.0, 0.5 M NaCl, 5% glycerol, 1 mM β-mercaptoethanol, 0.1 µM PMSF), cells were lysed by sonication (Misonix, 3000) on ice for 10 minutes. After centrifugation at 40,000*g* for 30 min, the clarified cell lysate was applied on a column of TALON metal affinity resin (Clontech). The column was washed with 10 column volumes of wash buffer (20 mM Tris pH 8.0, 0.5 M NaCl, 5% glycerol, 10 mM imidazole). Protein was eluted with 2 column volumes of elution buffer (20 mM Tris pH 8.0, 500 mM NaCl, 5% glycerol, 250 mM imidazole). Protein was further purified by gel filtration on a HighLoad 16/60 Superdex 200 column (GE Healthcare). Fractions containing protein were concentrated to a final value of 10 mg/ml, and stored in 20 mM Tris, pH 8.0, 500 mM NaCl, 5 mM DTT.

### Crystallization and structure determination

Crystals of the N-terminal CARD domain were grown at 18°C using the hanging drop method by mixing equal volumes of protein (10 mg/ml) and crystallization buffer containing 1.0 M K/Na tartrate, 0.1 M MES pH 6.0. The second Ig domain crystals were grown in 30% PEG 550-MME, 0.2 M Ammonium sulphate, and 0.1 M Sodium cacodylate at pH 6.5 in hanging drops. Crystals of the tandem Ig domains were grown at 18°C using the sitting drop method by mixing equal volumes of protein (10 mg/ml) and crystallization buffer containing 1.5 M MgCl_2_, 0.1 M Bicine, pH 9.0. Suitable crystals were cryoprotected by immersion in well solution supplemented with 15% (v/v) glycerol prior to dunking and storage in liquid nitrogen.

Diffraction data of the CARD domain, IgL2 and tandem Ig-like domains of MALT1 were collected at beamline F1 at the Argonne Photon Source, Beamline 19-ID; beamline F1 at the Cornell High Energy Synchrotron Source; beamline CMCF-1 at the Canadian Light Source, respectively. The data was processed with HKL-2000 [25] and then imported to the PHASER or into the SOLVE/RESOLVE programs [26]. Iterative manual model building using the graphics program Coot [27] and maximum-likelihood, maximum-entropy, and TLS refinement with REFMAC5 led to the final models. Parameters for Translation/liberation/screw (TLS) refinement were generated using the TLSMD web server [28]. Structures were deposited in the Protein Data Bank with codes 2G7R, 3BFO and 3K0W.

### Analytical Ultracentrifugation

Sedimentation equilibrium was carried out using a Beckman Optima XL-A analytical ultracentrifuge with an An-60 Ti rotor. Tandem IgL domains in 20 mM Tris-HCl pH 8, 200 mM NaCl and 1 mM TCEP was spun at 12,000 and 15,000 rpm at 4°C. Absorbance was recorded at 230 and 280 nm. Three sample concentrations were used for each analysis: 0.25, 0.5 and 1.0 mg/ml. Data analysis was performed using the Origin MicroCal XL-A/CL-I Data Analysis Software Package Version 4.0.

### Size exclusion chromatography

MALT1 domains at 10 mg/mL concentration in 50 mM Tris buffer, pH 8.0, 500 mM NaCl, 1 mM tris-(2-carboxyethyl) phosphine hydrochloride (TCEP) were loaded onto superdex-200 16/60 (GE healthcare) size exclusion column at 4°C. Standard proteins included thyroglobulin (670 kDa), globulin (158 kDa), conalbumin (75 kDa), ovalbumin (44 kDa), anhydrase (29 kDa), myoglobin (17 kDa), and ribonuclease (13.7 kDa).

## Supporting Information

Figure S1 **Electrostatic CARD surface.** Surface charge features of CARD domain of MALT1 (upper) and Bcl-10 model (lower). Two orientations are shown.
(TIF)

Figure S2 **Analytic ultracentrifugation.** Sedimentation equilibrium centrifugation analysis of the MALT1 tandem IgL1–IgL2 domains. The absorbance was measured at 280 nm at speeds of 12,000 rpm at 4°C. The lower panel shows the sedimentation equilibrium ultracentrifugation data for tandem IgL1–IgL2 domains at 1 mg/ml. Points shown were collected at 15,000 rpm and 4°C.
(TIF)

Figure S3 **Electrostatic surface of dimeric MALT1 tandem Ig-like domains.** (**A**) In the first dimer assembly of tandem Ig-like domains, two molecules were anti-parallel to each other in a cross-braced form. (**B**) The second dimer assembly of tandem Ig-like domains forms a tetramer. The electrostatic surface of the two tandem domains is presented in an open book style.
(TIF)

Datapack S1 Standalone iSee datapack - contains the enhanced version of this article for use offline. This file can be opened using free software available for download at http://www.molsoft.com/icm_browser.html.
(ICB)

Text S1 Instructions for installation and use of the required web plugin (to access the online enhanced version of this article).
(PDF)
